# Hemodynamic monitoring in the critically ill: an overview of current cardiac output monitoring methods

**DOI:** 10.12688/f1000research.8991.1

**Published:** 2016-12-16

**Authors:** Johan Huygh, Yannick Peeters, Jelle Bernards, Manu L. N. G. Malbrain

**Affiliations:** 1 ZNA Stuivenberg, Lange Beeldekensstraat 267, B-2060 Antwerpen, Belgium

**Keywords:** Hemodynamics, therapeutics, monitoring techniques

## Abstract

Critically ill patients are often hemodynamically unstable (or at risk of becoming unstable) owing to hypovolemia, cardiac dysfunction, or alterations of vasomotor function, leading to organ dysfunction, deterioration into multi-organ failure, and eventually death. With hemodynamic monitoring, we aim to guide our medical management so as to prevent or treat organ failure and improve the outcomes of our patients. Therapeutic measures may include fluid resuscitation, vasopressors, or inotropic agents. Both resuscitation and de-resuscitation phases can be guided using hemodynamic monitoring. This monitoring itself includes several different techniques, each with its own advantages and disadvantages, and may range from invasive to less- and even non-invasive techniques, calibrated or non-calibrated. This article will discuss the indications and basics of monitoring, further elaborating on the different techniques of monitoring.

## Introduction

Patients admitted to the intensive care unit (ICU) in general suffer from organ failure (single or multiple) or are at risk of such organ failure, which includes patients after major surgery and/or trauma. Hemodynamic instability, causing a mismatch between oxygen delivery and demand, is a major contributive factor for organ failure. Alterations in effective circulating volume (e.g. hypovolemia), cardiac function, and/or vascular tone (e.g. vasoplegic shock in sepsis) underlie hemodynamic instability ^1^. We can often manage it with regular clinical examination and monitoring of certain basic vital parameters (heart rate, blood pressure, central venous pressure [CVP], peripheral and central venous oxygen saturation, and respiratory variables) and urine output, but when these fail there is an increased need for hemodynamic monitoring (cardiac output [CO], pulmonary arterial occlusion pressure [PAOP or wedge pressure], pulmonary arterial pressure [PAP], mixed venous oxygen saturation [SvO _2_], stroke volume variation [SVV], extravascular water, etc.) to guide fluid management and vasopressor/inotropic support. Over the last few decades, hemodynamic monitoring has evolved from basic monitoring of CO to sophisticated devices providing a plethora of variables. These techniques and devices can be classified in either of two ways: 1) calibrated versus non-calibrated techniques and 2) by their degree of invasiveness (invasive, less invasive, or non-invasive). In this article, we will provide an overview of the indications and limitations for hemodynamic monitoring and the available methods of doing so.

## Indications for hemodynamic monitoring

All patients admitted to the ICU should be monitored, but the degree of monitoring can vary. Hemodynamically stable patients require maybe nothing more than continuous electrocardiographic (ECG) monitoring, regular non-invasive blood pressure measurement, and peripheral pulse oximetry (peripheral oxygen saturation or SpO _2_). Those who are unstable, or at risk of instability, should receive an arterial line for continuous invasive blood pressure measurement and regular analysis of arterial blood gasses. Any patient receiving vasopressors or inotropic agents requires a central venous line for drug administration and, when indicated, measurement of CVP and central venous oxygen saturation (ScvO _2_). When initial resuscitation fails to improve the hemodynamic and/or respiratory status of the patient, advanced hemodynamic monitoring will be required to guide medical management. Measuring CO and its components (preload, afterload, and contractility) will tell us if there is ongoing need for fluid resuscitation, vasopressors, or inotropic agents. It can be used as a diagnostic tool to determine the type of shock (hypovolemic, cardiogenic, obstructive, or distributive) according to the hemodynamic profile. Furthermore, it can be used to guide de-resuscitation, the phase after reconvalescence during which we are often confronted with fluid overload (in itself an important negative prognostic predictor) ^2, 3^. The clinical context (emergency room, operating room, or ICU) and the different possible variables provided by the monitoring method will determine which method we will use. There is, however, an important remark to be added when discussing indications for monitoring. Trials have as of yet not been able to show a significant reduction in mortality when comparing monitoring to standard of care, although there are possible benefits concerning complications ^4– 7^.

## Basics of hemodynamic monitoring

Measuring the CO starts with understanding the Fick principle, described by Adolf Fick in 1870 ^8^. In essence, this states that the blood flow to an organ can be calculated by using an indicator and measuring the amount of indicator that is taken up by the organ and its respective concentrations in arterial and venous blood. When we think of the entire human body as the organ described and use oxygen as the indicator, we can measure CO using this formula:

$$CO=\frac{VO_{2}}{CaO_{2}-CvO_{2}}$$

In this formula, VO _2_ is the consumption of oxygen and CaO _2_ and CvO _2_ are the arterial and mixed venous oxygen contents, respectively. The VO _2_ can be measured using a spirometer within a closed rebreathing circuit. Arterial and mixed venous oxygen are measured using blood samples from a peripheral arterial line (oxygenated blood) and a pulmonary artery catheter (PAC) (deoxygenated blood), respectively. This method is therefore invasive and time consuming, and although considered the gold standard it is rarely performed.

## Methods of hemodynamic monitoring

Several invasive and less-invasive methods have been developed during the last few decades to measure CO. The first to be used was the PAC, introduced in the 1970’s by Swan, Ganz, and Forrester ^9^. It is still the gold standard in the clinical setting to which we refer when comparing different methods of hemodynamic monitoring. These can be classified as calibrated or non-calibrated techniques or according to their level of invasiveness (invasive, less invasive, or non-invasive). There is a trend to use more less-invasive and non-invasive techniques to reduce the risks that accompany (less) invasive techniques.

Repeated calibration is performed in order to eliminate or reduce bias in continuous measurements. It refers to the act of evaluating and adjusting the precision and accuracy of the equipment. The precision of a technique is the degree to which repeated measurements (at the same time) show the same results, and the accuracy is the degree of closeness of the results to the actual true value (obtained by the gold standard method). Non-calibrated techniques try to reduce bias by implementing correction factors based on patient demographics (age, weight, gender, etc.) or calculations. However, in situations where preload, afterload, contractility, and aortic compliance can vary widely (as in critical illness), calibration will often prove necessary.

### Invasive techniques

***Pulmonary artery catheter (calibrated).*** The gold standard, the PAC, is a flow-directed catheter that is placed through an introductor in the jugular, subclavian, or, more seldom, the femoral vein and that travels from the right atrium through the right ventricle just until the pulmonary artery. It allows direct simultaneous measurement of pressures in the right atrium (CVP), PAP, and PAOP or wedge pressure, which in turn is indicative of the filling pressures in the left atrium. Blood sampling from the distal port (pulmonary artery) allows measurement of SvO _2_, and using fiber optic reflectometry allows for continuous monitoring of the SvO _2_. CO is measured with thermodilution. Initially, a cold saline bolus has to be delivered through the opening in the right atrium, with a thermistor detecting the drop in temperature a few centimeters from the tip of the catheter. Later, a heating coil is incorporated in the design, negating the need for cold fluid boluses (and thus avoiding bias because of different operators). This CO measurement, however, is not a true continuous monitoring seeing as it represents the average value of the last 5 minutes, and changes in CO during alterations in preload or afterload (e.g. fluid challenge) cannot be appreciated instantaneously. It also provides several calculated variables such as systemic and pulmonary vascular resistance, left and right ventricular stroke work, and the oxygen extraction ratio. Intracardiac electrodes allow the monitoring of electric activity, from which volumetric variables such as right ventricular ejection fraction (RVEF) and continuous assessment of right ventricular end diastolic volume (CEDV) can be gauged, providing information concerning right ventricular contractility and preload, respectively.

Although PAC was the most widely used technique in the past, a clear survival benefit has not been proven ^10^. The complexity of possible variations in obtained pressure tracings has led to large inter-observer variability, together with reports of very common misinterpretation of tracings ^11^.

The best indication for the PAC remains when there is right ventricular heart failure or pulmonary hypertension, seeing as no other monitoring device is capable of providing direct measurement of the pressures in the right heart and pulmonary circulation.

### Less-invasive techniques

***1. Transpulmonary thermodilution: the PiCCO ^®^ system (calibrated/surrogate gold standard).***

Using a central venous catheter and arterial line with thermistor, the PiCCO ^®^ system provides both intermittent (for calibration) and continuous CO measurement. The intermittent CO is measured using a transpulmonary thermodilution technique, where a cold fluid bolus is injected through the central line. Using the Stewart Hamilton equation, the area under the thermodilution curve is then used to calculate the CO. By using an algorithm based on the analysis of the arterial pulse contour, it is possible to continuously monitor CO and stroke volume, allowing assessment of beat-to-beat variations of stroke volume and CO in changing preload conditions. SVV and pulse pressure variation (PPV) have been proposed as variables to guide fluid loading in critical care settings ^12, 13^, although limited to completely sedated patients under controlled mechanical ventilation and in the absence of cardiac arrhythmias (LIMITS: low heart rate/respiratory rate ratio, irregular heart beats, mechanical ventilation with low tidal volume, increased abdominal pressure, thorax open, spontaneous breathing) ^14^.

Furthermore, the PiCCO ^®^ system allows the measurement of global end diastolic volume (GEDV), intrathoracic blood volume (ITBV), and extravascular lung water (EVLW). Pulmonary blood volume (PBV), pulmonary vascular permeability index (PVPI), global ejection fraction (GEF), contractility, and systemic vascular resistance (SVR) are derived from these values. These values can be indexed to body surface area and predicted body weight.

This system has several advantages over PAC: it is less invasive, it provides a true continuous CO and rapidly available measurements allowing the assessment of fluid responsiveness, and it is supported by literature data in humans that show good correlation between intermittent and continuous transpulmonary thermodilution CO with the PAC as gold standard.

Its drawbacks are the need for a specialized arterial line (typically placed in the femoral artery), a central venous line (jugular or subclavian vein), and regular calibration (three to four times a day) with cold fluid boluses (extra fluid load). The volume measurement is not automated and not continuous. It is less useful in valvulopathies, abdominal aortic aneurysm, or enlarged atria, and it is not applicable in arrhythmias or during intra-aortic balloon counterpulsation.

***2. Transpulmonary thermodilution: the VolumeView ^®^/EV1000 ^®^ system (calibrated).*** The VolumeView ^®^/EV1000 ^®^ system is similar to the PiCCO ^®^ system but differs in the measurement of the GEDV, where it uses a formula implementing the maximum upslope and downslope time of the thermodilution curve, whereas the PiCCO ^®^ system employs time constants derived from the mean appearance, mean transit, and downslope of the thermodilution curve ^15^.

***3. Transpulmonary dye dilution: the LiDCO ^®^ system (calibrated).*** Instead of thermal dilution, the LiDCO ^®^ system uses lithium as an intravascular indicator injected through a central or peripheral vein which is then measured in a peripheral artery using a specialized sensor probe attached to the pressure line ^16^. It is coupled to a pulse contour analysis system (LiDCOrapid ^®^/PulseCO ^®^). The only additional measured variables compared to PAC monitoring are the PPV and SVV. The data are rapidly available and provide real-time beat-to-beat variations in CO. Volume quantification, however, is not available, and the technique cannot be used in children/patients with a weight below 40 kg or patients under the influence of muscle relaxants (the positively charged quaternary ammonia ion is detected by the lithium sensor, affecting its measurements). Little is known about possible toxic effects or accumulation with long-term use of lithium. Furthermore, the ion-selective electrode is delicate and expensive and needs to be replaced every three days.

***4. Ultrasound flow dilution: the COstatus ^®^ system (calibrated).*** The COstatus ^®^ system calculates CO by using transpulmonary ultrasound dilution technology to measure changes in blood ultrasound velocity and blood flow following an injection of saline ^17^. It requires a primed extracorporeal arteriovenous tube set (AV loop) connected between the *in situ* standard arterial catheter and central venous catheter where two ultrasound flow-dilution sensors are placed on the arterial and venous ends. During calibration, a small roller pump is used to circulate blood through the AV loop from the artery to the vein. The ultrasound sensors provide an ultrasound dilution curve through which CO can be calculated following the Stewart Hamilton principle. After calibration, a continuous CO can be calculated through the arterial waveform. It calculates certain volumetric indices such as total end diastolic volume (TEDV), central blood volume (CBV), and active circulation volume (ACV), and it can detect intracardiac shunts. It is validated in both adult and pediatric patients. Recalibration is necessary in unstable conditions.

***5. Pulse contour and pulse pressure analysis (non-calibrated).*** Several devices use the technique of pulse pressure analysis to estimate CO. The difficulty is that, to estimate CO from pulse pressure analysis, one would not only need information about the heart rate and blood pressure but also have to make an estimate about the pressure-volume relationship of the aorta. Most of the techniques being used today are based on a three-element model integrating aortic characteristic impedance, arterial compliance, and systemic vascular resistance. These models work relatively well in stable patients but lack accuracy in unstable patients or when vasoactive drugs are employed ^18^. There are several devices using pulse pressure analysis available:

- FloTrac ^®^/Vigileo ^®^: a widely used method that uses PPV and vascular tone to calculate stroke volume and CO, although it is less useful in situations with low vascular tone (e.g. septic shock) ^19^.
- ProAQT ^®^/Pulsioflex ^®^: continuously measures CO by analyzing the systolic portion of the pressure wave after an initial autocalibration (depending on patient characteristics) or manually entering a starting cardiac index; there is, however, too large a percentage error ^20^.
- LiDCOrapid ^®^/pulseCO ^®^: uses the same algorithm as in LiDCOplus with calculating a nominal stroke volume from the entire pressure waveform. It can be calibrated using other techniques. There is, however, insufficient accuracy compared with thermodilution methods ^21^. Calibration improved this accuracy (even in critically ill patients) but only for the first four hours ^22^.
- Most Care ^®^/pressure recording analytical method (PRAM): uses an algorithm called “pressure recording analytical method”, which is a theoretical method developed by analyzing both pulsatile and continuous flow ^23^; only an invasive arterial catheter is needed, the implementation is easy, and it shows good accuracy in a wide range of settings.

***6. Respiratory derived cardiac output monitoring system: partial CO _2_-rebreathing (NiCO ^®^) (non-calibrated).*** Using CO _2_ instead of O _2_ as an indicator in the Fick principle (see above), the NiCO ^®^ uses a partial rebreathing method to measure the CO. The system consists of a CO _2_ and airflow sensor combined with a pulse oximeter. We can measure the CO _2_ production by multiplying the exhaled CO _2_ content by the respiratory minute volume. The arterial CO _2_ is derived from the end tidal CO _2_. Every three minutes, a partial rebreathing cycle should be started using a rebreathing loop, resulting in reduced CO _2_ elimination. By assuming CO is stable in both normal and rebreathing conditions, the difference between normal and rebreathing ratios are used to calculate CO. However, as it is dependent on stable ventilation, this can be used only in fully sedated patients with volume-controlled ventilation. Significant pulmonary disease (as in ICU patients with acute respiratory distress syndrome, pneumonia, atelectasis, shunting, etc.) can interfere with the measurements. To date, insufficient data exist to support its accuracy, specifically in critically ill patients.

***7. Transesophageal echocardiography (operator dependent).*** Transesophageal echocardiography (TEE) is an important cardiovascular diagnostic tool in perioperative and critical care medicine. It uses ultrasound to provide real-time images of the cardiac structures and blood flow. The transducer is placed in the esophagus next to the heart to produce these images. It may help define pathophysiological abnormalities in patients like wall motion abnormalities, pericardial effusions, pulmonary hypertension, and valvulopathy, in conjunction with other invasive or less-invasive monitoring. Guidelines published by the American Society of Anesthesiologists and the Society of Cardiovascular Anesthesiologists state that TEE should be used in critical care patients with persistent hypotension or hypoxia when diagnostic information expected to alter management cannot be obtained by transthoracic echocardiography (TTE) or other modalities in a timely manner ^24^. There is, however, a significant learning curve, TEE is expensive, and continuous monitoring is not an option. There is a (low) risk of oropharyngeal bleeding and dislocation of the endotracheal tube, and its use is relatively contraindicated in esophageal pathologies and severe coagulation abnormalities.

***8. Esophageal Doppler (operator dependent).*** Using a flexible ultrasound probe, the blood flow in the descending aorta is measured to determine stroke volume and CO. This probe can be left in place for prolonged periods of time (barring dislocation) and can provide real-time CO as well as afterload data interpretation. It provides many additional measurements as well as an estimate for preload via the corrected flow time. It is a promising, easy-to-learn technique associated with reduced hospital stay and better perioperative volume optimization ^25^.

### Non-invasive techniques

***1. Transthoracic echocardiography (operator dependent).*** CO can be measured with TTE using pulsed wave Doppler velocity in the left ventricular outflow tract (LVOT). It can also be measured at the mitral valve annulus, ascending aorta, right ventricular outflow tract (RVOT), and pulmonary artery, but these have been less validated. Seeing as there is less influence of systemic vascular resistance (SVR), measurements over the RVOT can provide an accurate CO, but only if there is no interference due to pulmonary arterial hypertension.

***2. Non-invasive pulse contour systems (non-calibrated).*** These systems strive to determine CO based on an arterial pulse pressure curve, which is estimated by a completely non-invasive technique.

- T-line ^®^: this system uses applanation tonometry (with a pressure sensor placed upon the radial artery) and an autocalibrating algorithm to estimate CO; it has an acceptable accuracy compared to calibrated pulse contour analysis ^26^ but needs more validation.
- ClearSight ^®^/Nexfin ^®^/Physiocal ^®^ system: this estimates blood pressure by a cuff wrapped around the finger and photoplethysmography to constantly adjust cuff pressure to keep the arterial diameter constant, thus creating a pulse pressure curve (Peñáz principle) used to estimate stroke volume, CO, SVV, and PPV; however, its accuracy still needs improvement and declines even further in patients with low CO, finger edema, hypothermia, or a high peripheral resistance ^27^.
- CNAP ^®^/VERIFY ^®^: this uses the same technique of photoplethysmography but corrects for changes in vasomotor activity with a specific algorithm; although a high percentage of error has been reported, this is markedly lower when using precalibration with the thermodilution technique ^28^. Further validation is needed.

***3. Bioimpedance (non-calibrated).*** Using skin electrodes, a small electrical current is applied. Changes in voltage over the circuit are then caused by changes in impedance and/or volume of the conducting tissues. Blood has a relatively low resistivity, and changes in intrathoracic blood volume have a high impact on impedance accordingly. With this assumption, we can postulate that changes in thoracic impedance are largely dependent on three components: a baseline impedance indirectly proportional to the thoracic fluid content, tidal changes in intrathoracic blood volume caused by respiration, and small changes caused by the cardiac cycle. The latter are primarily due to changes in aortic volume, which can be used to estimate stroke volume and CO ^29, 30^. However, it does have important limitations. The impedance is influenced by all changes in thoracic fluid composition such as lung edema and pleural effusions. Changes in systemic vascular resistance will influence the volume changes in the aorta and will therefore interfere with CO measurements.

***4. Estimated continuous cardiac output (esCCO ^®^) (non-calibrated).*** This is a non-invasive device estimating the CO with an algorithm based on patient characteristics and measurement of heart rate, peripheral oxygen saturation, and non-invasive blood pressure. With these measurements, a pulse wave transit time is determined and combined with the heart rate to estimate the CO. Although it has the advantage of being non-invasive, it remains a mere estimation of the CO. Studies suggest an unacceptable high deviation compared to validated methods ^31, 32^.

***5. Ultrasonic cardiac output monitoring (USCOM ^®^) (non-calibrated).*** Measuring the flow velocity in the aortic and pulmonary outflow tracts, USCOM ^®^ combines this with pre-calculated valve areas to estimate a CO. It has a short learning curve and has few procedural risks. There is, however, quite a proportion of unobtainable imaging, the proposed valve areas can differ significantly from the truth (specifically in elderly patients, patients who are critically ill, and patients with structural heart disease), and there can be a big difference between the estimated output and the calibrated reference value ^33– 36^.

## Conclusion

Critically ill patients are often hemodynamically unstable (or at risk of becoming unstable), and advanced hemodynamic monitoring is recommended in complex situations or in patients with shock who do not respond to initial fluid resuscitation. We are offered a wide variety of techniques that range from invasive to less invasive and even non-invasive. These techniques can be calibrated or non-calibrated. In Table 1, a schematic overview is given of the discussed techniques with their respective advantages and disadvantages. Calibrated techniques offer the best precision and accuracy, and the obtained values concerning CO, preload, afterload, and different other derived values are of significant value in the hemodynamic stabilization of critically ill patients. Relying on non-calibrated techniques can prove difficult in critically ill patients, where rapidly changing conditions in preload, vasomotor tone, and cardiac function can often lead to misleading results, with a risk of inappropriate medical management, under- or over-resuscitation, and subsequent organ dysfunction. They can be of value, however, in stable conditions, with less- or non-invasive techniques negating the possibility of complications due to more invasive techniques. Pulse contour analysis, in particular, with the added functional variables SVV and PPV, can be of significant value in the assumption that the patient is in regular sinus rhythm and fully sedated under controlled mechanical ventilation. As is so often required in the medical management of critically ill patients, we will have to balance the benefits and risks of the different techniques in the hope of achieving the best possible outcome for our patient. We recommend using calibrated techniques in the critically ill and unstable patients, preferring less-invasive techniques to more-invasive ones. A PAC, however, can be particularly useful in patients with significant cardiac dysfunction, specifically when concerning right ventricular dysfunction or pulmonary arterial hypertension. During de-resuscitation, the monitoring technique should be re-evaluated (and likewise when the patient deteriorates again), and non-invasive techniques should be used whenever possible instead of (less) invasive techniques. Non-invasive techniques can be combined with transthoracic/transesophageal echocardiography to provide valuable additional information.

**Table 1.** Overview of monitoring methods.

Method	Examples of commercial name	Calibrated or not	Major advantages	Major disadvantages
*Invasive methods*				
Pulmonary artery catheter		Calibrated	Direct measurements in right atrium and pulmonary circulation	Delay in determining CO, most invasive, and risks involved
*Less-invasive methods*				
Transpulmonary thermodilution	PiCCO ^®^ VolumeView ^®^/EV1000 ^®^ LiDCO ^®^	Calibrated	Intermittent and continuous CO, added variables	Need for specialized arterial and central venous line, LIMITS (PiCCO ^®^ system)
Ultrasound flow dilution	COstatus ^®^	Calibrated	Continuous CO, added variables, can detect intracardiac shunts	Requires AV loop
Pulse contour and pulse pressure variation	FloTrac ^®^/Vigileo ^®^ ProAQT ^®^/Pulsioflex ^®^ LiDCOrapid ^®^/pulseCO ^®^ Most Care ^®^/PRAM	Non-calibrated	Continuous CO	Lack accuracy in unstable patients or during use of vasoactive drugs
Partial CO _2_ -rebreathing	NiCO ^®^	Non-calibrated	No need for intravascular devices	Only in sedated patients under volume control ventilation, interference from pulmonary disease
Transesophageal echocardiography		Operator dependent	Real-time images of the cardiac structures and blood flow	Learning curve, (low) risk of complications
Esophageal Doppler		Operator dependent	Real-time CO and afterload data, added variables	Risk of dislocation
*Non-invasive methods*				
Transthoracic echocardiography		Operator dependent	Direct measurement of CO and visualization of cardiac structures	Ultrasound characteristics often suboptimal in ICU patients
Non-invasive pulse contour systems	T-line ^®^ ClearSight ^®^/Nexfin ^®^/ Physiocal ^®^ CNAP ^®^/VERIFY ^®^	Non-calibrated	Non-invasive, simple tool	Less accurate, needs more validation
Bioimpedance		Non-calibrated	Simple tool, providing data concerning CO and fluid overload	Changes intrathoracic fluid content and SVR influence measurements
Estimated continuous cardiac output ^®^	esCCO ^®^	Non-calibrated	Uses widely available variable to estimate CO	Is only estimate, inadequate accuracy
Ultrasonic cardiac output monitoring ^®^	USCOM ^®^	Non-calibrated	Short learning curve and only few risks	Only estimate, uses standard valve areas which can differ in patients

AV loop, arteriovenous fistula; CO, cardiac output; ICU, intensive care unit; SVR, systemic vascular resistance.
